# Statistics of Insanity
*"Statistics of Insanity; being a Decennial Report of Bethlem Hospital, from 1846 to 1855 inclusive." By W. Charles Hood, M.D., Resident Physician of Bethlem Hospital, &c.


**Published:** 1857-07-01

**Authors:** 


### Art. IV.—STATISTICS OF INSANITY.*

THE work before us is a valuable contribution to the science of Psychological statistics. Bethlem Hospital admits within its walls three classes of patients, viz., CURABLES, INCURABLES, and CRIMINALS. It is to the first of these classes that Dr. Hood directs his attention. He says it is his object to confine his observations to

"The statistical history of the patients admitted as *curables* into Bethlem Hospital during the ten years ending December, 1855," by noticing in succession the following subjects:—

1. Patients admitted as Curable.

2. Age.

3. Sex.

4. Education.

5. Religion.

6. Domestic Condition.

7. Social Condition.

8. Residence.

9. Apparent and Assigned Causes.

10. Duration of Disease before Admission.

11. Number of previous Attacks.

12. Time of Attacks.

13. State of the General Health.

14. Form of Insanity.

15. Treatment of Insanity.

16. Causes of Death and post-mortem appearances.

During the years extending from 1846 to 1855 inclusive, the number of patients admitted as curable into Bethlem Hospital was as follows:—

| | |
|---|---|
| Male . . . . . . . . . . . | 1066 |
| Female . . . . . . . . . . | 1663 |
| Total . . . . . . . . . | 2729 |

* "Statistics of Insanity; being a Decennial Report of Bethlem Hospital, from 1846 to 1855 inclusive." By W. Charles Hood, M.D., Resident Physician of Bethlem Hospital, &c.

| | | |
|---|---|---|
| CURED.—Male | . . . . . . . . . . . | 574 |
| Female | . . . . . . . . . . | 905 |
| Total | . . . . . . . . . | 1479 |
| Per centage, 54·19. | | |
| DIED. —Male | . . . . . . . . . . . | 76 |
| Female | . . . . . . . . . . | 98 |
| Total | . . . . . . . . . | 174 |
| Per centage of deaths, 6·37. | | |

*Aggregate of the* 100 *Years ending* 31*st December*, 1855.

| Admitted. | Cured. | Per cent. | Died. | Per cent. |
|---|---|---|---|---|
| 19,373 | 8341 | 43·05 | 1603 | 8.27 |

It would appear, by comparing the per centage of recoveries and deaths in various British, American, and Continental asylums, that 39·74 per cent. are the recoveries upon the numbers admitted, and 10 per cent. the deaths on the numbers resident. The following historical facts connected with the early history of this hospitals are of interest:—

"On the authority of Stow, who derived his information from Dr. Tyson, the Physician to the Hospital at that time, 1294 patients were admitted between the years 1684 and 1703; and of these 890, or about 2 in 3, were cured. But between the years 1784 and 1794, when 1664 patients were admitted, the number of recoveries was 574, or only a little more than 1 in 3. We next learn, from a report which Dr. Prichard obtained from Mr. Lawrence, ("A Treatise on Insanity," 1835, p. 141,) that the number of recoveries *increased* after the Hospital was removed to its present site. This report extends from 1819 to 1833. During this period 2445 patients were admitted; and 1124, or 1 in a little more than 2, were discharged cured."

In estimating the per cent. of recoveries either in Bethlem or St. Luke's Hospitals, Dr. Hood fairly states that—

"It is necessary to bear in mind the particular rules of the Institution, which are peculiar to it and St. Luke's. These regulations render ineligible all applicants who have been insane for more than twelve months; all who are afflicted with paralysis, epilepsy, or any other form of convulsive disease; all who have been discharged, *uncured*, from other Hospitals; and all aged and weak persons, and pregnant women. In addition to which, those who have not recovered at the expiration of a year after admission, are dismissed.* Rules so

* Although the rules of the Hospital limit the period of residence for patients on the Curable Establishment to one year, the sub-committee have the power of stringent must have considerable interest upon the number of recoveries and deaths; and it is interesting to inquire what that influence may be. At first it might be supposed that the number of recoveries ought to be increased by leaving out unsatisfactory and hopeless cases; but, on the other hand, many additional recoveries would undoubtedly be recorded if the uncured patients were not discharged at the end of twelve months: the effect, therefore, of the rules of this Hospital upon these statistics is not at all evident. That many patients would recover if they were allowed to remain in the Hospital for a longer time than twelve months is very evident, and that this is so, may at once be shown by a table which gives the experience of the Salpêtrière, under Esquirol, for a period of ten years."

Certainly this is the rational mode of estimating the ratio of cures in any asylum, public or private. We feel satisfied that many patients, discharged as cured, cannot properly be included in this class. We have reason to believe that several patients, dismissed from one of our Hospitals as cured, were admitted within a few weeks and months into other asylums in a very deranged state of mind. Great caution should be exercised in placing patients among those considered as cured, and we cannot be too careful in not confounding cases of temporary tranquillity and apparent freedom from delusion or hallucinations with those who have *bonâ fide* been restored to reason; without great caution in this respect, the statistical data of asylums will be valueless. A patient cannot properly be considered as cured, merely because the mental excitement under which he laboured on admission has subsided, and he no longer appears to be under the influence of any aberration of idea. Instances have come under our own observation of patients having been discharged as "cured," who have, in a few days after their discharge, been acutely insane. Again, as Dr. Hood fairly represents, no fair comparison can be instituted between Bethlem and St. Luke's, and other public or private institutions for the treatment of the insane. In both of these institutions the cases are picked and selected; in other words, they only admit within the wards of the asylum cases of insanity presenting the most favourable conditions for recovery.

It is therefore obvious, when we consider that, in other asylums, bad as well as good cases are admitted, that the statistics of recovery in an asylum like St. Luke's cannot, with any degree of fairness, be brought into juxtaposition with the statistics of recovery of other asylums. We have no hesitation in extending, on the recommendation of the Resident Physician, that time to fifteen or eighteen months, if the character of the complaint justifies the hope of recovery or improvement; and the committee so thoroughly recognise this advantage, that very few patients are discharged "uncured" who have not had the benefit of such extension.

saying, that if the medical superintendent of a well-conducted private asylum were permitted to exclude all cases presenting an unfavourable aspect, the portion of cures would be considerably enhanced.

It appears from Esquirol's table, that of 2005 patients—

"Who agreed in nothing except in being cases which were presumed to be curable, 604 recovered during the first year, 497 in the second year, 71 in the third, and 46 in the seven succeeding years. The numbers cured in the second year, as compared with those in the first year, are nearly as 5 to 6; sometimes even more patients were cured in the second year than in the first: thus, in 1809, 209 patients were admitted, and of these 48 were cured in the first year, and 64 in the second year; and again in 1810, when 190 patients were admitted, 48 were cured in the first year, and 51 in the second. Such being the case, it is at once evident that the number of recoveries must be greatly affected by a rule which limits the time for recovery to a single year.

"It is not easy to estimate how much the Hospital gains in the number of recoveries from the rules which exclude complicated and incurable cases; but we learn from Esquirol that 795 incurable cases, or cases considered as incurable, were admitted, between 1804 and 1813, into the Salpêtrière, which is open to all classes of patients; and that, during the same period, (as appears in the preceding table,) 2005 patients were admitted as curable, of whom 1218 were cured. Of these 1218 patients 604 were cured in the first year, and 614 in subsequent years. In order, therefore, to arrive at any conclusion as to the influence of the rules of Bethlem upon the number of recoveries in that Institution, it is necessary to compare the number of cases which are not affected in consequence of the rule which limits the time of residence to one year, with the number of incurable or doubtful cases which, by other rules, are excluded. These, taking the experience of the Salpêtrière as a basis of calculation, will bear the proportion of 614 to 795; hence it appears that the increased chances of recovery by extending the time of residence are not quite equal to the number of doubtful or incurable cases which are excluded by the rules. The number of recoveries in Bethlem are, therefore, somewhat augmented by the rules as they at present stand."

On the subject of mortality, Dr. Hood observes:—

"The mean annual mortality in English public Asylums, from their first establishment, up to about ten years ago, exclusive of Bethlem and St. Luke's, is estimated by Dr. Thurnam at 11·86 per cent.: viz., 'that of County Asylums for only paupers 13·88 per cent.; that of County Asylums receiving both private and pauper patients, 10·46 per cent.; that of Asylums for patients of different classes, supported wholly or in part by charitable contributions, 8·93 per cent. The mortality of seven Scotch Asylums has been 7·52 per cent.; and that of 10 Irish District Asylums, during the comparatively short time they have been established, 8·7 per cent. Extended inquiry and consideration appear to justify our concluding, that taking considerable periods of time, during which there have been no extraordinary disturbing circumstances in operation, in a mixed County Asylum, or in one for the middle and opulent classes, as well as paupers, a mortality which exceeds 9 or 10 per cent. is usually to be considered as decidedly unfavourable, and one which is less than 7 per cent. as highly favourable. In regard to Pauper Asylums, I believe we may conclude, under similar limitations, that a mortality which exceeds 12 or 13 per cent. is very unfavourable; and that one which is much less than 10 per cent. is highly favourable.' "

In considering the above facts, Dr. Hood considers there is no reason for dissatisfaction, and he finds the recoveries varying so high, 51·19 per cent., death so low, 6·37. It appears the aggregate experience of the one hundred years in Bethlem Hospital, ending 31st December, 1855, represents the cures 43·05 per cent., and the deaths as 8·27 per cent.

The liability to insanity is considered to be nearly twice as great from 30 to 40 as from 50 to 60, and much more than quite as great as at any age subsequent to 60. Dr. Hood says :—

"The largest number of patients admitted into the Retreat at York, (and these not less than one-third of the whole,) were admitted between the age of 20 and 30: and there was a gradual decrease in the numbers for each subsequent decennial period of life. More cases were also admitted into the Ohio Asylum between 20 and 30; and in this respect the experience of the American Asylum agrees with that of the Retreat. This is not to be easily explained. In America it is possible that the greater freedom in the mode of living amongst the rising generation may have much to do with the matter: but this consideration can scarcely apply to the Quakers who find their way into the Retreat. In the Quakers, perhaps, the explanation may be in the care which is taken of the community—a care which will single out a case as soon as the first symptom of the malady begins to be manifested, and which does not let poverty be any hindrance to the necessary treatment. At any rate there is no reason to doubt the general conclusion which is drawn by Dr. Thurnam from the whole body of evidence; and certainly the experience in Bethlem during the last ten years is in harmony with it. Thus, in our own table, the numbers admitted between 20 and 30, and between 30 and 40, are nearly the same; 739 being admitted in the former period, and 759, an increase of 20, in the latter: and after 40 there is a gradual decrease in the numbers for each quinquennial period, 284, 242, 204, 135, 110, 72."

According to Esquirol, the greatest number of cures were from the 25th to the 30th year, and from the 30th to the 35th year; and that they go on progressively diminishing from the 45th year to the end of life—the diminution being more uniform in men, and more abrupt in women. Recovery, however, may take place at later periods of life; and these very tables show that twenty men recovered after the 50th year, of whom four were upwards of 70.

Speaking of this subject, Dr. Hood observes :—

"According to our own table the recoveries under 25 amount to about three-fifths of the admissions, and to about one half, between 30 and 65, if we neglect certain inconsiderable fluctuations. After 65, as might be expected, the recoveries are greatly diminished, being about one-seventh. This will be seen on referring to the table.

"The influence of age upon the number of deaths has also been carefully investigated. In our own tables the mortality, as a rule, increases rapidly with the age. Under 20, it is 4·8 per cent.; between 20 and 25, 2·5 per cent.; between 25 and 30, 3·9 per cent.; between 30 and 35, 4·5 per cent.; between 35 and 40, 8·4 per cent.; between 40 and 45, 5·6 per cent.; between 45 and 50, 7·8 per cent.; between 50 and 55, 7·8 per cent.; between 55 and 60, 8·1 per cent.; and above 60, 16·9 per cent. The mortality, as a rule, increases with the age; but under 20 it is higher than in the decennium following, and between 35 and 40 it is much higher than in the years immediately preceding and following; a curious fact, which cannot be easily explained."

Chapter III. of Dr. Hood's work, on "sex," contains much interesting and valuable information. He says truly that

"Esquirol investigated the subject very carefully, and concluded that women were a little *more* subject to insanity than men, the proportions being about 38 females to 37 males."

Dr. Thurnam, however, proved that he erred in his calculations in forgetting that the proportion of adult females, in the general population, exceeds that of the males. The excess is 12 per cent. from the age of 20 to 30, 6 per cent. from 30 to 40, and 4 per cent. from 40 to 50. He also erred in comparing the *existing*, instead of the *occurring*, cases of insanity in the two sexes. This would have been a matter of no moment if the progress of the disease was the same in the two sexes, but such is not the case. The number of recoveries is greater in women than in men; and the number of deaths is nearly 50 per cent. higher in men than in women. It is therefore evident, that to compare the simple number of cases existing at any one time, would give no true result; and we must take the cases *occurring*, and not the cases existing, if we would arrive at any correct conclusion respecting the comparative liability of men and women to insanity. Dr. Thurnam was the first to direct attention to this subject; and his conclusion, after a very careful examination of the evidence, was, that men are a little more liable to insanity than women. In the principal hospitals for the insane in these kingdoms he shows, "the proportion of men admitted is nearly always higher, and in many cases much higher, than that of women ; and as we know that the proportion of men in the general population, particularly at those ages when insanity most usually occurs, is decidedly less than that of women, we can have no grounds for doubting that the male sex is actually more liable to disorders of the mind than the female."

Again, in former years more women than men were admitted into Bethlem, as well as into St. Luke's, and the present data are in harmony with past experience:—1663 women having been admitted into Bethlem during the last ten years, and 1066 men, *i.e.*, 64 per cent. more women than men.

Dr. Hood considers that the

"Influence of sex upon recovery is supposed to be *very* marked; and it is generally agreed that the probability of recovery is *much* greater in women than in men. But this is not the conclusion which is to be drawn from the experience of Bethlem during the 10 years under consideration, for this experience shows that 907 out of 1663, or 54·4 per cent., recover among the women, and 574 in 1066, or 53·8 per cent., among the men—a difference in favour of the women, it is true, but far more inconsiderable than that which is usually supposed to exist.

"On the other hand, it is admitted that insanity is much more likely to end in *death* in men than in women. The mortality among men, indeed, has been supposed to be nearly double that among women; and this is a very remarkable fact, for the excess in the general mortality is not more than 5 or 6 per cent. on the side of the males. In our own tables the mortality among the men is considerably higher than among the women, but not to the extent of being double. It is 7·3 per cent. among the men, and 5·8 per cent. among the women."

In reference to the religious persuasion of patients admitted from 1845 to 1856 into Bethlem Hospital as curable, the following statistics are given:—

| | Males. | Females. | Total. |
|---|---|---|---|
| Church of England . . . . | 763 | 1251 | 2014 |
| Roman Catholic . . . . . | 44 | 55 | 99 |
| Wesleyan . . . . . . . . | 55 | 86 | 141 |
| Dissenter . . . . . . . . | 204 | 271 | 475 |
| | 1066 | 1663 | 2729 |

Dr. Hood next considers the domestic condition of patients admitted as curable during the same period (from 1846 to 1855 inclusive).

*Domestic Condition of Patients admitted as Curable.*

From 1846 to 1855 inclusive.

| | Admitted. | | | Discharged. | | | | | | | | |
|---|---|---|---|---|---|---|---|---|---|---|---|---|
| | | | | Cured. | | | Uncured. | | | Died. | | |
| | M. | F. | T. | M. | F. | T. | M. | F. | T. | M. | F. | T. |
| Married . . | 545 | 819 | 1364 | 302 | 448 | 750 | 123 | 236 | 359 | 53 | 59 | 112 |
| Single . . . | 475 | 719 | 1194 | 244 | 399 | 643 | 169 | 265 | 434 | 21 | 30 | 51 |
| Widowed. . | 46 | 125 | 171 | 28 | 58 | 86 | 5 | 41 | 46 | 2 | 9 | 11 |
| | 1066 | 1663 | 2729 | 574 | 905 | 1479 | 297 | 542 | 839 | 76 | 98 | 174 |

Dr. Prichard considered that

"The condition of married life is, *cæteris paribus*, much less liable to the excitement of madness than that of celibacy. The proportion of married and unmarried persons in the Salpêtrière and Bicêtre, during the 20 years ending 1822, according to a report by M. Desportes, was as follows:—

| | Females. | Males. |
|---|---|---|
| Unmarried . . . . . | 980 | 492 |
| Married . . . . . . | 397 | 201 |
| Widowers and Widows . | 291 | 59 |
| Divorced . . . . . . | 5 | 3 |
| Not noted . . . . . . | 53 | 9 |
| Total . . . | 1726 | 764 |

"Dr. Prichard also refers to Dr. Jacobi's *statistics* to show that the case is the same in Germany, thus:—

| | Females. | Males. |
|---|---|---|
| Unmarried . . . . . | 599 | 974 |
| Married . . . . . . | 156 | 176 |
| Widowed . . . . . . | 80 | 30 |
| Total . . . | 835 | 1180 |

How, asks Dr. Hood, are these numbers accounted for?

"'Is it,' Dr. Prichard asks, 'through the restraints which the condition of celibacy imposes, or through the vices to which unmarried persons are more frequently abandoned? M. Esquirol is of opinion that where one case of insanity arises from the former cause, a hundred result from the latter.' Again: 'we must take into our calculation, that married persons lead, in general, more regular lives in all respects than the unmarried; that they are for the most part, more fixed in their pursuits and in their condition as to maintenance and employment; and that they are in a less degree subjected to causes which agitate the mind and excite strong emotions. These remarks, however, apply principally to men, and the difference observed in respect to numbers is almost equally great among females.'

"Let the explanation be what it may, the conclusion must certainly be, that marriage does not 'predispose to insanity;' that marriage, in short, is a natural condition. At the same time, it must be remembered, that 'many of the cases of insanity among unmarried persons occur in a class, who, as regards bodily and mental vigour, are less likely to be married than the average of the community at large; so that in such cases the celibacy must be regarded as an *effect*, rather than as a cause of the condition predisposing to insanity.' (Thurnam *Op. cit.*, p. 72a.)

"It is more than probable, however, that more extended inquiries may alter materially the aspect of the case as it now stands. Thus the experience of Bethlem Hospital, during the last ten years, does not support the idea that unmarried persons are more likely to become insane than the married; on the contrary, the married patients were more numerous than the unmarried, in the proportion of 1364 to 1194. The question must therefore remain in abeyance for the present; and in the meantime we may notice the manner in which the chances of recovery or death are affected, or appear to be affected, by the domestic condition of the patient. We may not attach much importance perhaps to any such deduction, but it is curious to know that these chances are not the same in the married, unmarried, and widowed state; thus among the recoveries, we find 55·7 per cent. of the married, 53·8 per cent. of the unmarried, and 50 per cent. of the widowed; and among the deaths, we find 8·2 per cent. of the married, 4·2 per cent. of the unmarried, and 6·4 per cent. of the widowed."

With regard to the influence of social position in inducing insanity, Dr. Hood correctly says, the data are far too scanty to allow the formation of any sound opinion; and all that we can do is to notice a few salient points which present themselves on a cursory inspection of the column. It is curious, then, to notice that the medical men are nearly twice as numerous as the clergymen and lawyers, both of whom are equal in numbers; and yet this, perhaps, is what we might expect, when we consider the broken rest of the great majority of men of the medical profession: for if this broken rest is sufficient to shorten the average duration of their lives appreciably, it must also tell very perniciously upon their mental health. Nor is it surprising that the number of schoolmasters and musicians should be so high. Under the head of schoolmasters are a large number of those generally unfortunate persons called "tutors;" which, no doubt, is a sufficient reason why schoolmasters, as a class, swell the list so considerably, for the unsatisfactory social position in which tutors are too often placed tends necessarily to fret and irritate their minds. Musicians, on the contrary, themselves more excitable than the majority of the population, may be in danger from being made "too much of," by that part of society into which they are constantly welcomed. The number of clerks is high, though not higher, perhaps, than the extent of this class would lead us to expect. Comparing the number of those engaged in *sedentary mechanical in-door pursuits*, with those engaged in *non-sedentary* mechanical in-door pursuits, we do not find any very marked difference, but the preponderance is with the latter. Among the former, the shoemakers are most numerous, and then the tailors; among the latter, are first the carpenters (including the cabinet-makers), and then the bakers. These facts are curious, explain them as we may.

Among the female patients, the only points which seem to require notice, are the very large number of governesses and dressmakers (including milliners and sempstresses). It is no wonder that an elegant, accomplished, and otherwise delicately nurtured lady, should pass from unhappiness to misery, and from misery to insanity, in a position which too often is not half so desirable as that of a domestic servant; and of the causes which operate upon thousands of the class of dressmakers, who are driven mad by penury, trouble, and perhaps remorse, it is not necessary to speak.

With reference to residence, the following facts are of interest:—

| | Males. | Females. | Total. |
|---|---|---|---|
| London and its immediate neighbourhood . . . | 421 | 594 | 1016 |
| The Provinces . . . . . | 636 | 1048 | 1684 |
| Not ascertained . . . . | 9 | 20 | 29 |
| | 1066 | 1663 | 2729 |

The chapter on the assigned causes of insanity is replete with valuable matter. In speaking of hereditary predispositions, Dr. Hood remarks:—

"In the Bethlem tables the total number attributed to this cause simply, is 270 in 2729, or 10·28 per cent. among the women; and 8·3 per cent. among the men. It appears also that these cases are more unsatisfactory than the others, in so far as the chances of recovery are concerned, and less unsatisfactory in the chances of death. Thus, while the per centage of recoveries in both sexes is 51·5 where the cause of the disorder was of a moral character, and 33·8, where the cause was of a physical character, the per centage was only 14·6 where the only cause that could be detected was hereditary predisposition; and again, while the per centages of deaths in the cases of insanity arising in moral and physical causes are 62·5 and 24·3 respectively, the per centage is only 11·8 where the disorder was simply due to hereditary predisposition.

"There is no opportunity in Bethlem of calculating the influence which the hereditary tendency to insanity has upon the liability to relapse, but there is every reason to believe that this influence is very unfavourable."

The following extract from Dr. Hood's work, relating to the *moral* causes of insanity, embracing—1. Anxiety and Distress, 2. Uncontrolled Passions and Emotions, 3. Perverted Religion, we will not attempt to abridge.

The *physical* causes of insanity are considered at length by Dr. Hood, viz., 1. Injury to the Head; 2. Disease of the Nervous System; 3. Fever; 4. Intemperance; 5. Intestinal Disorder; 6. Physical causes peculiar to Females.

Dr. Hood enters at length into all these important points. We make no apology for quoting at length from this portion of his volume:—

"The experience of Bethlem, as gathered from the tables of the ten years under consideration, shows that the cases originating in moral causes are nearly double those originating in physical causes; the numbers being 980 to 571 in 2727. It also shows that the chances of recovery are greater, and the chances of death also greater, in cases originating in *moral* causes; thus the mean per centage of recoveries in cases arising from moral causes, is 51·5, and of deaths, 62·5; whereas the mean per centage of recoveries in cases arising from physical causes is 33·8, and of deaths, 24·3. It is also curious to learn that the chances of recovery are greater, and of death also greater, in the case of men becoming insane from moral causes, the numbers being 55·3, and 74·6; whereas the women have slightly the advantage, though very slightly, where the insanity has been induced by *physical* causes.

"*Anxiety and Distress*, in their multiform aspects, appear to be the grand causes of insanity; and in the tables of Esquirol they form considerably more than one-half of the entire number of the category of moral causes. In the Bethlem table 60·2 per cent. among the men, and 70·9 per cent. among the women, may be classed more or less directly under these heads. It is very doubtful, moreover, whether insanity ever arises from causes of an opposite nature, as from excess of joy. Indeed Esquirol has the remark, that the excess of joy which destroys life never takes away the reason; and he sets himself to explain away certain cases which are supposed to support a contrary conclusion. In answer to a statement of Mead, that fortunes rapidly acquired produce insanity in England—he asks, for instance, whether the persons thus becoming lunatic may not have become so in consequence of laying aside their former habits for idleness and luxury, and so on. He says, moreover, that no case of insanity which could be fairly attributed to excess of joy, has fallen under his own notice, and he mentions two cases in illustration of the mistake. A minister informs his relative of his nomination to an important place, and this relation immediately fell into a state of hypochondriacal melancholy—joy was thought to be the cause of this misfortune, but the real cause proved to be *despair* at having to quit a mistress. A young man gains a prize in a lottery, and a few days afterwards he was seized with insanity; excessive joy was thought to be the cause, but the real cause proved to be the *fear* of losing his treasure. Certainly it is no argument to the contrary, that insanity originates occasionally in 'sudden prosperity,' as in the six cases in the Bethlem tables; for here, *ennui* and many other analogous causes may have combined to unhinge a mind accustomed to action, and not trained to enjoy the '*otium cum dignitate*.' At any rate, nothing is known of these cases to contradict the *dictum* of Esquirol.

"*Uncontrolled Passions and Emotions.*—Arguing from the statistics of Esquirol, Dr. Prichard considers that the uncontrolled passions and emotions deserve to rank next to anxiety and distress in causing insanity, but this opinion is scarcely borne out by the Bethlem tables. Jealousy is certainly not an unfrequent cause; thus we find in the ten years under consideration the insanity of 5 men and 10 women referred to it: neither is fright an unfrequent cause, particularly among the women, for 48 cases in 545 among the women, and 4 in 435 among the men, are attributed to fright; but the numbers are not so high as the statement of Dr. Prichard would lead us to expect.

"*Perverted Religion.*—The remarks which belong to this head have been anticipated on a former page; (pp. 30, &c.,) and here it only remains for us to notice the number of the cases ascribed to this cause; which are, 37 in 435 among the men, and 11 in 545 among the women. These numbers are high, but we doubt very much whether they would not be even higher, if more was known of the real history of the Bethlem patients.

"*Physical Causes.*—These causes have been thought to act more powerfully upon women than upon men, and the Bethlem tables do not contradict this idea. The difference, however, does not appear to be very great, for the per centage among the men is 19·8, and among the women 21·5.

"*Injuries to the Head.*—Accidents of this kind, as Dr. Prichard says, are more frequently causes of delirium than insanity; but instances sometimes occur in which insanity is the consequence, delirium being the intermediate link. In the Bethlem tables 17 cases among the men, and 5 among the women, are referred to 'concussion.'

"*Diseases of the Nervous System.*—It is not easy to estimate the importance of these diseases, as causes of insanity; epilepsy is no uncommon cause, but we have no authentic data to determine the degree of frequency. The same remarks apply also to paralysis. Insanity is often referred to insolation, or coup-de-soleil; a condition which acts by exciting inflammation, or a state akin to inflammation, in the encephalon. The heat of the kitchen fire acts in the same manner occasionally upon cooks; coup-de-soleil, indeed, and 'coup-de-feu,' as it may be called, are frequently mentioned in Esquirol's tables, and they occur not unfrequently in the Bethlem tables, in which 11 cases, all among the men, are referred to coup-de-soleil.

"*Fever.*—There is no doubt that the foundation of insanity may frequently be traced to the delirium of typhus; and that the mental malady is often connected with a metastatic inflammation of the brain and its membranes, connected with rheumatism or gout. At the same time it is not less true, that active fever and insanity must be regarded as antagonistic conditions rather than otherwise. Galen cites a case of insanity which was terminated by a quartan fever; and Belgarrie states a similar fact. M. Esquirol also tells us that he has known several instances of insanity terminated by fever, either continued or intermittent (*Op. cit.*, p. 57). Where insanity is connected with fever, it is generally by the suppression of certain cutaneous eruptions, as of small-pox, &c.

"In the Bethlem tables, the cases referred to 'fever,' are 15 among 1066 men, and 12 among 1663 women; while the cases referred to 'rheumatism,' are 8 among the men, and 4 among the women.

"*Intemperance.*—Intemperance holds a high rank among the physical causes of insanity, as set forth in the Bethlem tables: the numbers under this head being 90 out of 212 among the men, and 40 out of 359 among the women. This contrasts unfavourably with the experience of M. Esquirol, who says, that among 336 lunatics staying in his own establishment, there were only three whose derangement was ascribable to this cause; but there is every reason to believe that intemperance is far more frequently the cause of insanity in this country at the present time, than was the case in France in the days of Esquirol.

"*Sensuality.*—Here again as in the case of intemperance in stimulating drinks, it is very difficult to arrive at any correct conclusion, for want of accurate data. In the Bethlem tables, however, the mental disorder is referred to 'onanism,' in 12 cases, and to 'sensual excess' in 11 cases. M. Esquirol says that one-twentieth of the lunatics in the Salpêtrière had been prostitutes. But it is a question whether grief, and anxiety, and broken hours, may not have had a greater share in dethroning the reason than sensuality.

"*Intestinal Disorders.*—Dr. Prichard lays great stress upon intestinal disorder as a frequent cause of insanity. 'The state of the intestinal canal,' he says, 'to which I allude, is itself much more frequently of an inflammatory nature than it has generally been imagined, or at least, than it was formerly supposed to be. In that condition of the canal which gives rise to costiveness, alternating with diarrhœa, and accompanied with indigestion, flatulence, and eructations, anorexia and nausea, transient but often acute pains in the hypochondria, livid and yellow suffusions of the skin, viscid secretions in the mouth, or redness of the fauces and palate with a glazed and dry surface; the whole train of symptoms often depends upon a low degree of chronic inflammation in the mucous membrane of the intestinal canal; and this is perhaps a frequent, if not an ordinary state in those cases in which disorders of the nervous system supervene in complaints of the stomach and bowels.'

(*Op. cit.*, p. 206.) This disorder may originate in various ways, but generally in errors of diet. Worms can very rarely be traced as a cause of insanity.

"Dr. Prichard's opinion, however, is scarcely borne out by the Bethlem tables, inasmuch as the cases referred to 'dyspepsia,' are comparatively few; 14 among the men, and 5 among the women. It is not at all improbable, however, that intestinal disorder has very often been overlooked by the persons supplying the past history of the patient; indeed, the subsequent history of the patient often renders it certain that this is the case.

"*Physical Causes peculiar to Females.*—Arguing from the history of *hysteria*, we are at once prepared to expect that uterine disorder in one form or another, will prove to be a frequent cause of insanity; and such is the fact. When the process of menstruation is insufficient and painful, there are often, as is well known, symptoms which may be said to foreshadow insanity; an irritable and quarrelsome disposition, a marked waywardness, a disposition to despond, and so on; and these symptoms are still more marked where the menses are altogether suppressed: amenorrhœa, there is reason to believe, is frequently one of the causes of insanity; certainly, the menses are often suppressed in insanity, and their re-appearance is often contemporaneous with recovery.

"The importance of uterine disturbance and hysteria, as physical causes of insanity, is well shown in the Bethlem tables; for in 359 cases, 76 are referred to these causes. It is also more than probable that uterine disturbance, or hysteria, has something to do with the cases ascribed to puerperal mania, and over-lactation; and if so, then these numbers are greatly increased, and instead of being 76 in 359, they will be 238 in 359, or 63·2 per cent.

"The only general conclusion which can be drawn from a consideration of the causes of insanity, is, that they are more or less obviously of an exhausting or depressing character; a conclusion which shows indirectly, what is now generally allowed, that insanity is a disease of depression, exhaustion, and irritation."

Here we must pause, reserving for another article the continuation of our analysis of Dr. Hood's essay.

---

